# Increased Adsorption of Heavy Metal Ions in Multi-Walled Carbon Nanotubes with Improved Dispersion Stability

**DOI:** 10.3390/molecules25143106

**Published:** 2020-07-08

**Authors:** Carolina Rodríguez, Sebastián Briano, Eduardo Leiva

**Affiliations:** 1 Departamento de Ingeniería Hidráulica y Ambiental, Pontificia Universidad Católica de Chile, Avenida Vicuña Mackenna 4860, Macul, Santiago 7820436, Chile; cnrodriguez@uc.cl (C.R.); sabriano@uc.cl (S.B.); 2 Departamento de Química Inorgánica, Facultad de Química, Pontificia Universidad Católica de Chile, Avenida Vicuña Mackenna 4860, Macul, Santiago 7820436, Chile

**Keywords:** multi-walled carbon nanotubes, dispersion, heavy metal removal, adsorption

## Abstract

In recent years, carbon nanotubes (CNTs) have been intensively studied as an effective adsorbent for the removal of pollutants from wastewater. One of the main problems for its use corresponds to the agglomeration of the CNTs due to the interactions between them, which prevents using their entire surface area. In this study, we test the effect of dispersion of oxidized multi-walled carbon nanotubes (MWCNTs) on the removal of heavy metals from acidic solutions. For this, polyurethane filters were dyed with a well-dispersed oxidized MWCNTs solution using chemical and mechanical dispersion methods. Filters were used in column experiments, and the sorption capacity increased more than six times (600%) compared to experiments with suspended MWCNTs. Further, kinetic experiments showed a faster saturation on MWCNTs in column experiments. These results contribute to a better understanding of the effect of dispersion on the use of CNTs as heavy metal ions adsorbent.

## 1. Introduction

In the last decades, there has been a growing concern about the effect of high concentrations of heavy metal (loids) in the environment. Heavy metal (loids) can contaminate freshwaters with devastating effects on the aquatic ecosystems and can have a toxic effect on human health [1,2,3,4]. For this reason, the development of technologies for the removal of heavy metals (loids) from contaminated water has become an important challenge due to their persistence and recalcitrance in the environment.

The increase in industrial production in the last decades is the main source of heavy metal in wastewaters, air, and soils [5,6]. In northern Chile, mining industries are important sources of heavy metal (loids) that decrease water quality in several rivers [7,8,9]. Particularly, in the northern regions of Chile, mining activity uses more than 50% of the water of this zone and is responsible for the generation of wastewater characterized by a low pH and high concentrations of heavy metals [10,11,12]. This contamination is known as acid mine drainage (AMD), and some of the main heavy metals (loids) found in it are iron (Fe), copper (Cu), manganese (Mn), zinc (Zn), arsenic (As), among others [13,14,15].

In this context, the removal of heavy metal (loids) from contaminated water has become the focus of the development of new technologies where the use of nanomaterials and nanoparticles has shown significant progress. Nanoparticles have many advantages for heavy metal adsorption processes, among them a large surface area per unit mass (specific surface) and high reactivities [16,17]. In addition, a small number of nanoparticles can treat a large volume of water due to its high mobility in aqueous solutions [18].

The study of new carbon structures dates back to the mid-1980s with the laboratory synthesis of the fullerene (C_60_), a 60 carbon atom superstable structure with never-before-seen characteristics, including its interaction with different types of compounds, such as oxygen and transition metals [19]. Starting from this, a large number of studies were carried out, so much so, that in 1991, multi-walled carbon nanotubes (MWCNT) were synthesized for the first time [20] and in 1993 single-walled carbon nanotubes (SWCNT) [21] with similar characteristics to those reported for fullerene but with other new ones of great importance in the biological, electrical, and environmental fields [22]. This new allotropic form of carbon is made up of cylindrical sheets of graphite rolled into a tube [23]. SWCNTs are those that only have a graphite shell rolled up in a cylindrical form [21], while MWCNTs are made up of multiple layers of graphite sheets [5,20,24].

Recently there have been numerous articles on the adsorption properties of carbon nanotubes highlighting their ability to remove various heavy metals due to its accessible external surface, low mass density, well-developed mesopores (pores between 2 and 50 nm), and an easily modifiable surface [25,26]. This last characteristic is one of the most developed to maximize its absorption capacity of heavy metals [27,28,29]. A large number of surface modification techniques have been studied for this nanomaterial, one of them being acid treatment. It has been shown that the acid treatment can be performed with different types of acids such as HNO_3_, NaOCl, H_2_SO_4_, HCl, among others [27,30]. This type of technique allows introducing different oxygenated functional groups (carboxylic groups) onto the surface of CNTs, which improves their ability to absorb heavy metal by new mechanisms, especially by electrostatic interactions between metal ions towards the modified surface of CNTs [5,31]. The treatment could be carried out successfully due to the hexagonal arrangement of the carbon atoms in each graphite tube, which allows the surface to generate strong interactions with other molecules, even with other CNTs [32].

Other benefits of surface modification using acids are the removal of impurities from untreated CNTs and the higher availability of more adsorption sites [5]. In fact, adding oxygenated functional groups to the surface of the CNT gives them a certain hydrophilic character, which improves the dispersion of the material in aqueous solution due to electrostatic repulsion [23]. Because of mutual attraction, pure CNTs tend to agglomerate in the presence of water due to van der Waals interactions [5,33]. Agglomeration is a problem when working with this type of material, since it makes it impossible to use the entire surface area available for the sorption process, causing large losses in efficiency due to this phenomenon that can be partially solved with the acid treatment [25,31,34].

In this matter, current studies demonstrate the effectiveness of CNTs as adsorbents of different water pollutants for various conditions of pH, contact time, temperature, and adsorbent mass [18,35,36]. However, there are still issues to investigate and improve, such as the large-scale production of CNT [5,36,37] or surface modifications that do not use large amounts of chemicals that may be harmful to the environment [5,18,36]. CNTs can cause cytotoxic effects on microbes and affect aquatic life [38]. Some in vivo studies have shown that CNTs induce lung cell proliferation and lung inflammation [39]. However, the toxicity of CNTs depends on several factors. In general, agglomerated CNTs are more toxic than well-dispersed CNTs [5,40]. In the same line, it is necessary to study treatment techniques for the regeneration of CNTs after water treatment [5,36]. To address this, the use of adsorption columns can be a proper alternative, where the process can be more economical, efficient, and simple compared to batch studies for heavy metal removal, partly because CNT separation after use in a batch process can be more complex and expensive [35].

Our previous study [41] showed that the oxidation processes of MWCNTs improved their adsorption capacity for heavy metal removal. Using nitric acid (HNO_3_) as an oxidizing agent, the surface oxygen content increased from 4.8% to 14%. Additionally, better dispersion of oxidized MWCNTs in water was observed. However, the tendency of the MWCNTs to agglomerate prevents them from exhibiting their total surface area. With this motivation, the aim of this study was to explore the effect of oxidized MWCNTs dispersion from preliminary treatment column experiments, using a support dyed with a widely dispersed MWCNT ink. The relevance of this work lies in the quantification of the effect of an optimal dispersion of MWCNTs in the removal of contaminants and the preliminary evaluation of the design of a treatment system.

## 2. Results and Discussion

### 2.1. Characterization of MWCNTs

**Figure 1:**
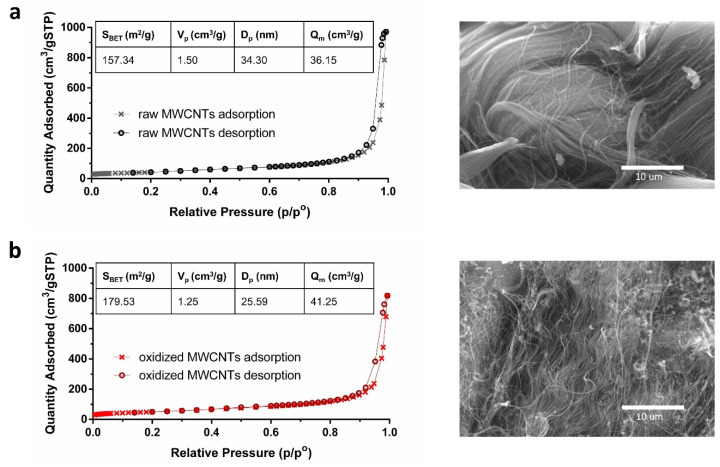
BET adsorption-desorption profiles and SEM images for (**a**) raw MWCNTs and (**b**) oxidized MWCNTs. S_BET_: BET surface area; V_p_: pore specific volume; D_p_: pore diameter; Q_m_: maximum adsorption capacity.

Characterization using Brunauer–Emmett–Teller (BET) analysis (Figure 1) showed an increase in the BET surface area (S_BET_) and the maximum sorption capacity (Q_m_) of the MWCNTs after the oxidation process. Furthermore, a decrease in pore specific volume (V_p_) and pore diameter (D_p_) was observed. This same behavior has been reported by other studies [42,43,44] using different oxidizing agents such as nitric acid, hydrogen peroxide, and potassium permanganate. Similarly to our results, Li et al. [44] showed an increase from 122 to 154 m^2^/g in S_BET_ with CNTs treated with HNO_3_. The increase in S_BET_ and Q_m_ suggests that oxidized MWCNTs have better potential as an adsorbent material than raw MWCNTs. Additionally, the adsorption isotherms of raw and oxidized MWCNTs exhibited similar patterns to type III and type V adsorption isotherms according to the International Union of Pure and Applied Chemistry (IUPAC) classification, indicating the presence of relatively weak adsorbent-adsorbate interactions [45]. A hysteresis loop type H3 was also observed, characterized by having a very steep adsorption branch near saturation. In general, the loops of this type are given by non-rigid aggregates of plate-like particles [45].

The images obtained by SEM showed a clear structural change in the MWCNTs surface after oxidation (Figure 1). The MWCNTs tips were partially opened by the action of the HNO_3_, and structural defects were generated due to the attachment of oxygen-containing groups [46,47].

**Figure 2:**
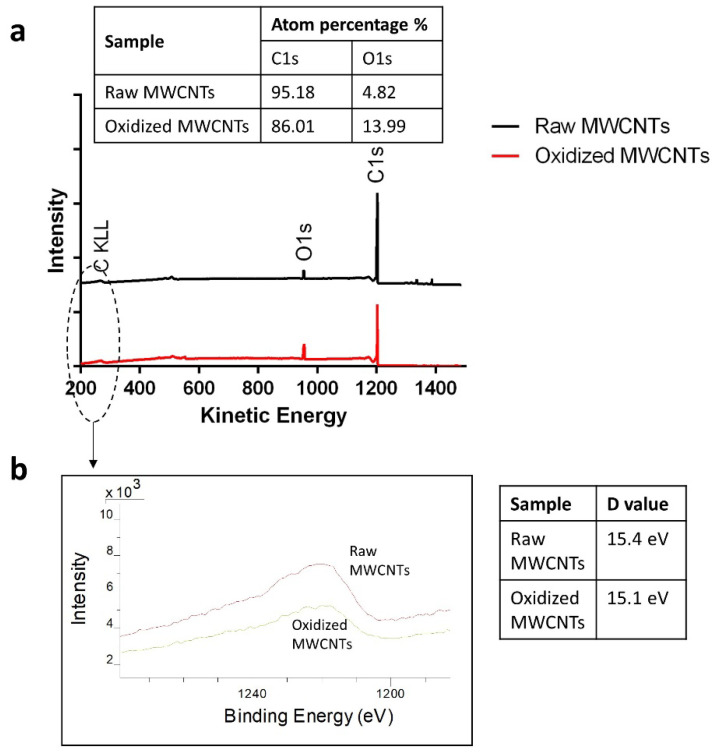
XPS analysis of (**a**) complete spectra of raw and oxidized MWCNTs and (**b**) the auger region and parameter D values.

Superficial analyzes using XPS showed an increase in the oxygen percentage (Figure 2). The samples analyzed showed that the surface oxygen content increased from 4.82 to 13.99% after the oxidation process. Other studies show similar percentages in the oxygen content after chemical oxidation [48,49,50]. Some researchers have tested with stronger oxidation processes, which are capable of introducing more oxygen-containing groups such as 34% [41] or 22% [51] of the total mass, but the strong action of oxidizing agents could seriously damage the structure of MWCNTs [52,53]. Additionally, the auger region was studied to evaluate the sp^2^/sp^3^ content from carbon KLL spectra (C KLL). For this, the parameter D was determined, corresponding to the distance between the most positive maximum and the most negative minimum of the first derivate of the C KLL spectra [54]. For both, raw and oxidized MWCNTs, the D value was close to 15 eV, so the oxidation process did not significantly affect the sp^2^/sp^3^ hybridization ratio. The two small signals on the right-hand side of the primary XPS region of carbon (C1s) in the raw MWCNTs spectrum are attributable to the siloxane signals characteristic of the silicon wafer used to support the samples.

### 2.2. Qualitative Analysis of Dispersion

**Figure 3:**
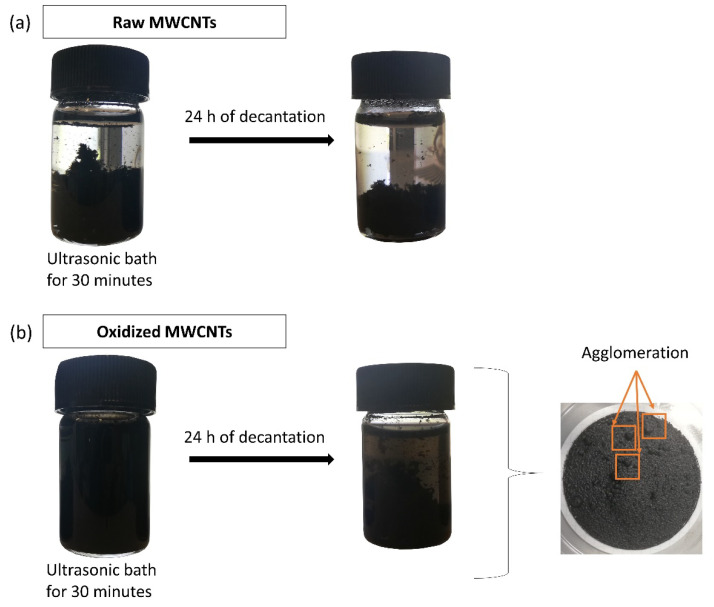
Dispersion experiments of (**a**) raw and (**b**) oxidized MWCNTs. Photographs were taken after 30 min of dispersion in an ultrasonic bath and after 24 h of decantation.

Raw and oxidized MWCNTs were dispersed by sonication to observe their affinity and dissemination in an aqueous matrix. In this experiment, the effect of two different dispersion methods was observed. The first is a chemical method corresponding to oxidation that enhances wetting or adhesion characteristics. The second is a mechanical method that separates CNTs from each other but can also fragment them, increasing their aspect ratio [55,56]. Figure 3 shows photographs after 30 min of dispersion and after 24 h of settling. It is possible to observe that the raw MWCNTs were not significantly affected by sonication, remaining agglomerated and easily distinguishable within the solution. Conversely, the oxidation process and the increase of oxygen-containing groups improved the dispersion in water. After 24 h, the oxidized MWCNTs had decanted, but a fraction remained in suspension. The decanted fraction was part of the excess of the added MWCNTs, while another part remained well-dispersed in the solution. Moreover, the oxidized MWCNTs were recovered using a 0.22 μm membrane filter. As shown in Figure 1b, some agglomerations are distinguished. The undispersed MWCNTs powders cannot exhibit their total surface area [31].

Several studies have reported that the oxidation of CNTs can increase their dispersion and solubility in water, forming well-dispersed electrostatically stabilized colloids in water and other organic solvents [57,58,59]. Oxygen-containing groups introduced during the oxidation process formed stronger interactions with the aqueous solvent than solvent/solvent and MWCNTs/MWCNTs interactions, then the oxidized MWCNTs were preferentially enclosed by solvent molecules, which resulted in repulsive interactions between the MWCNTs in the solution that stabilized them against reaggregation [60]. Sezer and Koç [61] reported that MWCNTs treated with HNO_3_ exhibited excellent dispersion stability with no visual sedimentation for three months. Besides, Lee et al. [62] studied the dispersion of modified CNTs bearing carboxylic acid (CNT-COOH) and carboxylic anion (CNT-COO^−^), which showed stable dispersion for more than two months in aqueous solution, while pristine CNTs settled down after one day. Therefore, oxygen-containing groups introduced during the oxidation process significantly improve the dispersion in polar solvents [63].

### 2.3. Effect of the Adsorbent Mass

**Figure 4:**
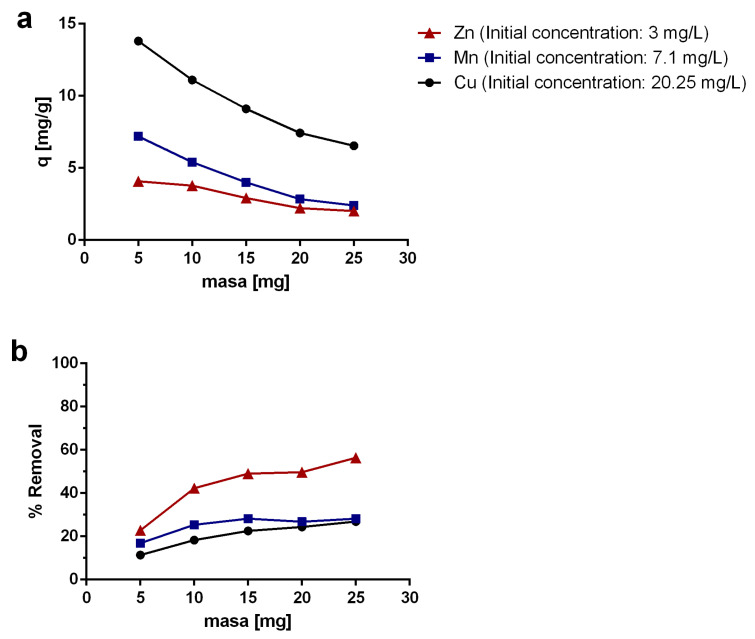
(**a**) Sorption capacity (q) [mg/g] and (**b**) removal rate of Cu, Mn, and Zn. Experiments were carried out using a constant adsorbate concentration and a variable mass of oxidized MWCNTs as sorbent material.

Since the raw MWCNTs were not effective for metal adsorption [41], oxidized MWCNTs were used for the analyzes and experiments. Adsorption experiments were carried out for different masses of oxidized MWCNTs (5–25 mg), using solutions with constant volumes and metal concentrations. The results for Cu, Mn, and Zn showed that the sorption capacity q (mg/g sorbent) decreases progressively with increasing adsorbent mass (Figure 4). This can be explained because CNTs have a strong tendency to agglomerate and to form aggregates due to van der Waals interactions [5]. Thus, the interactions between CNTs would be promoted with a greater mass of oxidized MWCNTs in a confined space, where a progressive increment in the amount of MWCNTs increases the probability of interaction between MWCNTs particles. Higher mutual attraction forces between CNTs cause them to stick together, thereby reducing their exposed surface [33]. Particularly, this effect is critical for heavy metal removal processes, which depends in part on the availability of the affinity sites on the surface of the CNTs. Therefore, even though the removal rates are higher with increasing MWCNTs mass, the amount of metal removed per gram of adsorbent decreases. Very few studies have reported the effect of the CNTs mass as a function of the sorption capacity (mg/g sorbent). Dehghani [64] investigated the phenol removal using SWCNTs and MWCNTs. They reported that the removal rate increased with increasing adsorbent mass but not linearly, and over the 100 mg of sorbent, the removal became constant. These results correlated with that obtained in this study, in which the sorption capacity was affected by increasing the amount of adsorbent in a constant volume.

For Cu, the results showed that the sorption capacity decreased 52.6% when 25 mg of oxidized MWCNTs was used instead of 5 mg. In the case of Mn and Zn, the decrease was 66.6% and 50.6%, respectively. It can be seen that the removal efficiency increased with increasing MWCNTs mass. However, a better dispersion should improve the sorption capacity of the oxidized MWCNTs in the studied mass range.

### 2.4. Column Experiments

For this experiment, new dispersion methods were added. In addition to oxidation and ultrasonic bath, ultrasonication was used as a stronger mechanical method. Besides, a surfactant (SDBS) was added to improve the dispersion stability after separation. Li et al. [65] showed that the adsorption capacity using the SDBS surfactant was almost pH-independent for pH values lower than 9.0, while the adsorption capacity using other surfactants such as octyl-phenol-ethoxylate (TX-100) and benzalkonium chloride (BKC) is strongly affected by the pH. Even though in our study it was shown that chemical oxidation improved dispersion, after 24 h, a fraction of MWCNTs had decanted. For this reason, surfactants are very useful for obtaining more stable dispersions over time. Some studies have shown that surfactant-dispersed CNTs can be colloidally stable for weeks [66,67].

The sorption capacity was determined for a filter dyed with an oxidized MWCNTs ink. The filter was used in column experiments for water treatment. These results were compared with those obtained in the adsorption experiments with powdered (suspended) MWCNTs using monometallic and multimetallic waters. For all experiments, the adsorbent mass used was approximately 20 mg. To avoid MWCNTs particles release during the column experiment, the filters were dyed, dried, and washed with deionized water successively until a constant mass was obtained. It should be noted that only about 30% of the ink used for each filter adhered to the polyurethane. To compare the experiments appropriately, the results are summarized in Table 1. It can be seen that the column experiments showed a better sorption capacity compared to the results of the suspended MWCNTs. For Cu, the sorption capacity was increased by around six times (600%) using treatment columns instead of suspended MWCNTs for multimetallic waters. In the case of Mn and Zn, the increase was even greater, reaching an increase of 667% and 4300%, respectively.

Although adsorption assays with powdered nanotubes were carried out after dispersing the samples with an ultrasonic bath for 30 min, it was observed that the oxidized MWCNTs tend to agglomerate and decant in time (Figure 1). Previous studies have reported the effect of the dispersion of CNTs on the capacity of sorption [31,34]. Undispersed CNTs powders cannot exhibit their total surface area, so well-dispersed CNTs may show a greater capacity of sorption for metals than undispersed CNTs based solely on the surface area available for sorption [31]. This could explain the significant increase in the sorption capacity for oxidized MWCNTs in column experiments compared to suspension experiments. The same effect was reported by Wang et al. [68], who used calcium-alginate beads impregnated with functionalized MWCNTs for the removal of methylene blue, where column experiments allowed greater dispersion stability in the treatment filters. They observed that the sorption capacity increased considerably compared to suspended MWCNTs. Likewise, Khan et al. [69] studied the removal of cadmium and bismarck brown R with modified MWCNTs and also obtained better results using treatment columns. They found that the best results in the column experiments could be due to the higher adsorbate concentration onto the adsorption zone, whereas in batch experiments, the adsorbate concentration decreases with time gradient.

For the design of our column experiments, polyurethane filters (commercially known as a sponge) were used because they allow the flow of water through them and do not show interactions with the heavy metals to be removed. This was tested with a control experiment using an undyed sponge, where no removal of Cu, Mn, and Zn was observed (Data not shown). Many authors [31,68] have reported that the main limitations for a large-scale treatment system are to ensure that the CNTs remain fixed on the filters, to avoid agglomeration with other particles, and to prevent the release of CNTs in the treated water, as this would cause serious damage to the environment and the human health. Several studies have reported the possible effects of CNTs on human health and aquatic ecosystems [70,71,72]. Due to the size of the nanoparticles, they can enter cells directly through the cell membrane and cause mainly lung and inflammatory diseases [73,74,75,76]. These limitations are also evidenced in our work, in which MWCNTs nanoparticles that were not retained by the filter were released during the preparation phase of the experiment. Ensuring that the filters only contain well-retained nanoparticles on their surface is a critical step in the design of a treatment system, as well as the potential for filter reuse. Previous studies, including one own work, studied the desorption process as a function of pH in powdered CNTs [29,41,77,78]. In general, at low pH, good desorption rates were obtained. However, there are few desorption studies in filter treatment systems. Thus, the use of a proper and optimized matrix for the CNTs allows recycling the filter for subsequent uses and operations [18].

### 2.5. Kinetics Experiments

**Figure 5:**
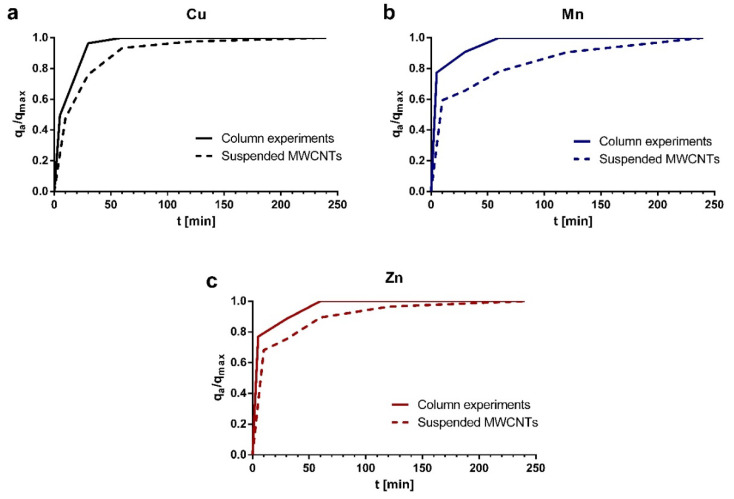
Accumulated sorption capacity (q_a_) divided by maximum sorption capacity (q_max_) of (**a**) Cu, (**b**) Mn, and (**c**) Zn using polyurethane filters dyed with oxidized MWCNTs ink in column experiments (continuous lines) and suspended oxidized MWCNTs in batch experiments (dashed lines).

To compare the kinetics of the experiments performed on columns and with suspended MWCNTs, the results are presented as the accumulated sorption capacity (q_a_) over time, divided by the maximum sorption capacity (q_max_) reached at equilibrium (Figure 5). In column and batch experiments, the equilibrium was reached approximately at 1 and 2 h, respectively. It is observed that suspension experiments take longer to reach equilibrium. This could be explained due to MWCNTs in column experiments are more dispersed and exposed so that saturation occurs faster, while the suspended MWCNTs constantly interact with each other, and saturation is slower. Kinetic experiments show another advantage of the design of treatment columns with continuous flow compared to batch treatments, since it requires a shorter contact time for the removal of contaminants [79].

## 3. Materials and Methods

### 3.1. Characterization of MWCNTs

The raw and oxidized MWCNTs were characterized by Brunauer–Emmett–Teller analysis (BET) (Micromeritics Instruments Corp., Norcross, GA, USA), Scanning Electron Microscopy (SEM) (JSM-IT300LV, JEOL Ltd., Tokyo, Japan), and X-ray photoelectron spectrometry (XPS, SPECS FlexMod, Berlin, Germany), using MWCNTs dispersed in deionized water on a silicon wafer.

### 3.2. MWCNTs Oxidation

The MWCNTs were commercially acquired from NanoTechLabs, Inc. (Yadkinville, NC, USA). The oxidation process was carried out by reflux at 120 °C for 4 h using 500 mg of raw MWCNTs in 250 mL of 65% nitric acid, according to the method described in Rodríguez and Leiva [41]. The obtained solution was filtered using a 0.45 µm membrane filter and washed with deionized water until reaching a neutral pH. The filtrate was dried at 60 °C overnight.

### 3.3. Qualitative Analysis of Dispersion

Samples of raw and oxidized MWCNTs were dispersed in deionized water using an ultrasonic bath (Isolab Laborgerate GmbH, Wertheim, Germany) for 30 min. Then, the samples were allowed to decant for 24 h. Photographs were taken during the process to observe the dispersion of the MWCNTs.

### 3.4. Adsorption Experiments

Adsorption experiments were performed using 20 mg of oxidized MWCNTs with 30 mL of metal ion solution. For this, different concentrations of Cu (~20 mg/L), Mn (~6 mg/L), and Zn (~3 mg/L) were prepared, as in our previous study [41]. The solutions with oxidized MWCNTs were shaken for 14 h at 350 rpm and room temperature (~25 °C). The metal concentrations in the aqueous phase were measured colorimetrically in a Hach Spectrophotometer DR/2010. The procedure was carried out for monometallic and multimetallic solutions.

The sorption capacity *q* (mg/g sorbent) for each experimental condition was obtained using the equation

(1) $$q=\frac{\left(C_{0}-C_{e}\right)V}{m}$$

where:

- C_o_: initial concentration of metal in aqueous solution (mg/L).
- C_e_: equilibrium concentration of metal in aqueous solution (mg/L).
- V: total volume of solution (L).
- m: the mass of sorbent (g).

### 3.5. Effect of the Adsorbent Mass

Adsorption experiments were performed using different masses of oxidized MWCNTs (5, 10, 15, 20, and 25 mg). The adsorbent was mixed with 30 mL of a monometallic solution: Cu (~20 mg/L), Mn (~6 mg/L), and Zn (~3 mg/L). The solutions were shaken at 350 rpm for 14 h and subsequently filtered using a 0.45 µm membrane filter. The metal concentrations in the aqueous phase were measured colorimetrically in a Hach Spectrophotometer DR/2010.

### 3.6. Column Experiments

**Figure 6:**
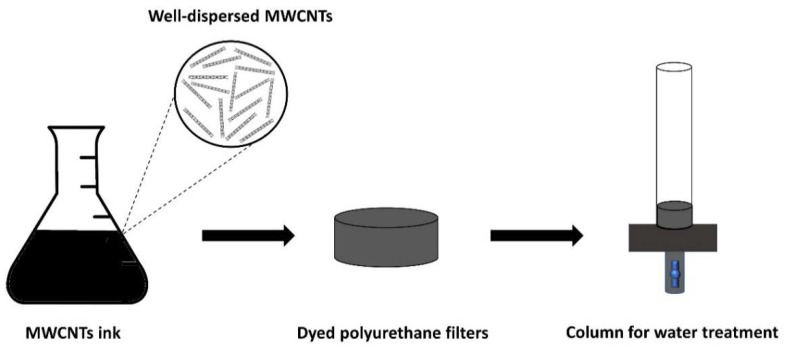
Schematic diagram for sorption experiments using a water treatment column.

Treatment columns were designed to evaluate the dispersion effect using polyurethane filters dyed with oxidized MWCNTs ink. The ink was prepared by adding oxidized MWCNTs (1.5 mg/mL) and the surfactant sodium dodecylbenzenesulfonate (SDBS) (10 mg/mL) to deionized water. The mixture was sonicated in an ultrasonic bath (Isolab Laborgerate GmbH, Wertheim, Germany) for 15 min and ultrasonicated using a Fisherbrand™ sonicator Q500 (Fisher Scientific, NH, USA) for 30 min. This oxidized MWCNTs ink has a high dispersion due to the use of ultrasonication. The polyurethane filters were immersed for 5 min in the nanotube ink, then were washed with deionized water and dried at 60 °C for 12 h. This staining process was carried out twice. The number of nanotubes present in each filter was calculated by mass difference before and after staining. Then, continuous flow sorption experiments were carried out to evaluate the metal removal over time and determine the maximum sorption capacity of the nanotubes on a well-distributed support. Sorption experiments were performed on acrylic columns (diameter: 4 cm; height: 40 cm) using continuous flow (30 mL/min). The experiments were carried out using multimetallic water with metal ion content, as described in 3.3. Figure 6 shows a scheme of the experiment.

## 4. Conclusions

Our results showed that the oxidation using HNO_3_ increased the surface area and the nitrogen sorption capacity of MWCNTs, according to BET analysis. The insertion of oxygen-containing groups improved the MWCNTs dispersion, but it is still possible to observe agglomerations of oxidized MWCNTs. The ultrasonication and impregnation of oxidized MWCNTs on a support dramatically improved the heavy metal removal. The column experiments with oxidized MWCNTs in polyurethane filters showed an increase of more than 600% for the removal of Cu, Mn, and Zn compared to batch experiments. These findings contribute to a better understanding of the main limitations of CNTs’ use in large-scale treatment systems and the effect of some techniques commonly used to disperse CNTs on the sorption capacity.

Even so, additional efforts should be made in the research and development of appropriate support material for the use of CNTs in large-scale treatment systems.

## Figures and Tables

**Table 1:** Comparison of sorption capacities of oxidized MWCNTs in batch experiments (suspended MWCNTs) and columns (filters).

	Sorption Capacity q (mg/g Sorbent)		
	Cu^2+^	Mn^2+^	Zn^2+^
Column experiments (MWCNTs on a support (filter), multimetallic water)	35.2 ± 1.8	9.2 ± 0.8	4.4 ± 0.6
Competition experiments (suspended MWCNTs, multimetallic water)	5.0 ± 1.0	1.2 ± 0.2	0.1 ± 0.05
Adsorption experiments (suspended MWCNTs, monometallic water)	7.3 ± 0.6	2.8 ± 0.1	2.2 ± 0.1

Average data are shown with standard deviation.
